# Multiplication of photonic band gaps in one-dimensional photonic crystals by using hyperbolic metamaterial in IR range

**DOI:** 10.1038/s41598-023-27550-2

**Published:** 2023-01-06

**Authors:** Aliaa G. Mohamed, Walied Sabra, Ahmed Mehaney, Arafa H. Aly, Hussein A. Elsayed

**Affiliations:** TH-PPM Group, Physics Department, Faculty of Science, Beni-Suef University, Beni-Suef, 62521 Egypt

**Keywords:** Materials science, Optics and photonics, Physics

## Abstract

The light-slowing effect near band endpoints is frequently exploited in photonic crystals to enhance the optical transmittance. In a one-dimensional binary photonic crystal (1DPC) made of hyperbolic metamaterials (HMMs), we theoretically examined the angle-dependent omnidirectional photonic bandgap (PBG) for TM polarization. Using the transfer matrix approach, the optical characteristics of the 1DPC structure having dielectric and HMM layers were examined at the infrared range (IR). As such, we observed the existing of numerous PBGs in this operating wavelength range (IR). Meanwhile, the HMM layer is engineered by the subwavelength dielectric- nanocomposite multilayers. The filling fraction of nanoparticles have been explored to show how they affect the effective permittivity of the HMM layer. Furthermore, the transmittance properties of the suggested structure are investigated at various incident angles for transverse magnetic (TM) and transverse electric polarizations. Other parameters such as, the permittivity of the host material, the filling fraction of nanoparticles, and the thickness of the second layer (HMM) are also taken into account. Finally, we investigated the effect of these parameters on the number and the width of the (PBGs). With the optimum values of the optical parameters of the nanocomposite (NC) layer, this research could open the way for better multi-channel filter photonic crystals.

## Introduction

Photonic crystals (PCs) have received a lot of interest since their invention because of their ability to control light propagation^1–3^. One key characteristic of PCs is the photonic bandgap (PBG) which prevents light propagation in its waveband^4, 5^. Because of their unique features, PCs are utilized to create optical devices such as reflectors^6–8^, lasers^9, 10^, fibers^11, 12^, and tunable optoelectronic devices^13–15^. In optical systems, omnidirectional reflectors by using PCs are crucial research point^6, 7, 16^. The PBG of PCs can be created through dielectric films; however, it is influenced by the angles of incidence. Notably, the propagation phase in the dielectric materials is reduced with increasing the incidence angle. Because of the iso-frequency curve of dielectric materials being circular or elliptical, the PBG is shifted toward the shorter wavelengths. As a result, there are few uses of PCs with blue-shifted PBGs in the production of omnidirectional reflectors^6, 7, 16^.

Numerous investigations into omnidirectional broadband PBGs have been demonstrated^17–20^. In this regard, metamaterials^21^ are utilized to create PCs with a variety of PBGs^22–24^ and have exceptional electromagnetic characteristics. The zero-average index gap in PCs composed of negative-index metamaterials and alternative dielectric materials is omnidirectional and unaffected by the incident angle^19, 25^. In PCs containing different permittivity- and permeability-negative metamaterials, the zero effective phase gap was angle-independent^26, 27^. Recently, hyperbolic metamaterials (HMMs) have a lot of interest^28–35^. HMMs are considered as a kind of substantially anisotropic metamaterials that can be used to create narrow-band absorbers, broadband reflectors, near-field heat radioactive heat transfer, and super-resolution imaging^28–35^. Contrary to normal dielectric materials, HMMs propagation phase increases with increasing the incident angle because their iso-frequency curve is hyperbolic. By using the HMM and traditional dielectric material phase-variation compensating effect can produce new features such as angle-independent and red-shifted PBGs^36, 37^. As reported by the authors in Ref.^38^, if the propagation phase for the two band edges of the PBG is opposite in a unit cell with a HMM and dielectric layer, the PCs can display a novel angle-dependent omnidirectional broadband PBG with red-shifted and blue-shifted edges for the long-wavelength and short-wavelength bands, respectively.

Ma. et al.^39^ discuss the properties of HMs composed of the plasma and ordinary medium and after their parameter adjustment, the developed structure realizes single-frequency reflection in absorption (SFRA) with angle stability. In our paper, we will discuss the properties of HMM composed of dielectric and nanocomposite material where we obtained many PBGs in our structure in IR range with less angle stability. Also, PCs arranged in one-dimensional lattice have fewer complex structures and they are often easier to be constructed than two- and three-dimensional nanostructures. Therefore, in this study, we investigate the omnidirectional PBG in one-dimensional PC designs containing the HMMs.

Thus, the basis of the Maxwell–Garnett models and the well-known characteristic matrix method serve as the mathematical basis for our theoretical investigations. We pay more attention on the transmittance properties of this one-dimensional PC design. Wherein, further studies into the optical properties of the suggested structure is carried out at various HMM layer thicknesses, the effective permittivity of host material, the filling fraction, and the incident angle for transverse magnetic (TM) and transverse electric (TE) polarizations. It is worth mentioning that TE wave is referred as transverse electric wave mode. On the other side, TM wave is referred as transverse magnetic wave mode. TE mode is also known as H mode as there is only a magnetic field along the direction of propagation. Whereas TM mode is also known as E mode as there is only an electric field along the direction of propagation.

## The theoretical analysis of one-dimensional PBG in PCs having HMMs

**Figure 1:**
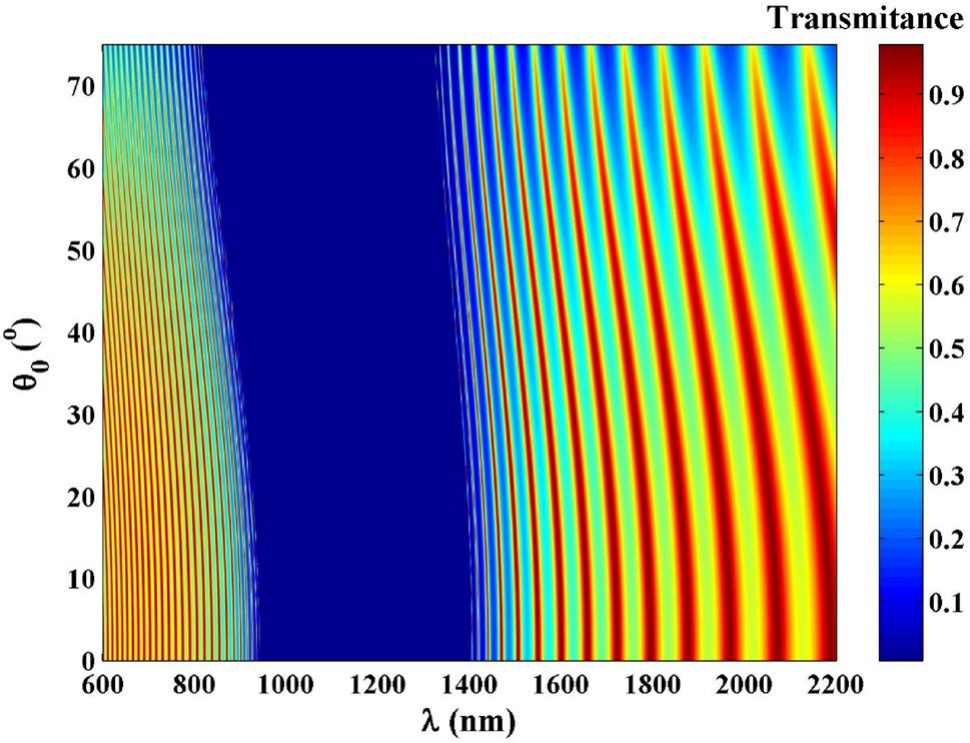
The colour map for the PC design ${\left(\mathrm{AB}\right)}^{30}$ for TM polarization.

Here, we introduce an omnidirectional PC $\left( {AB} \right)^{N}$, in which two media (A and B) are designed for N periods. In this design, layer A is considered as $\left( {{\text{InAs}}} \right)$ with refractive indices, $n_{A}$ and thickness $,d_{A}$. Then, layer B is the nanocomposite material which composed of Ag nanoparticles embedded in host material (Yttrium oxide) such that $\left( {{\text{Ag}}:{\text{Y}}_{2} {\text{O}}_{3} } \right)$. For this layer, the refractive index and thickness are defined as $n_{B}$ and $d_{B}$, respectively. By using the transfer matrix approach^40^, the transmission spectra of the proposed PC design $\left( {AB} \right)^{30}$ with changing the incident angles are shown in Fig. 1 where only the transverse magnetic (TM) waves are considered and the magnetic field (H) direction is kept parallel to the surface of the proposed PC design. As the incident angle is increased, the PBG edges are shifted to low wavelengths like the traditional one-dimensional PC. Such response is due to Bragg conditions^6^.

**Figure 2:**
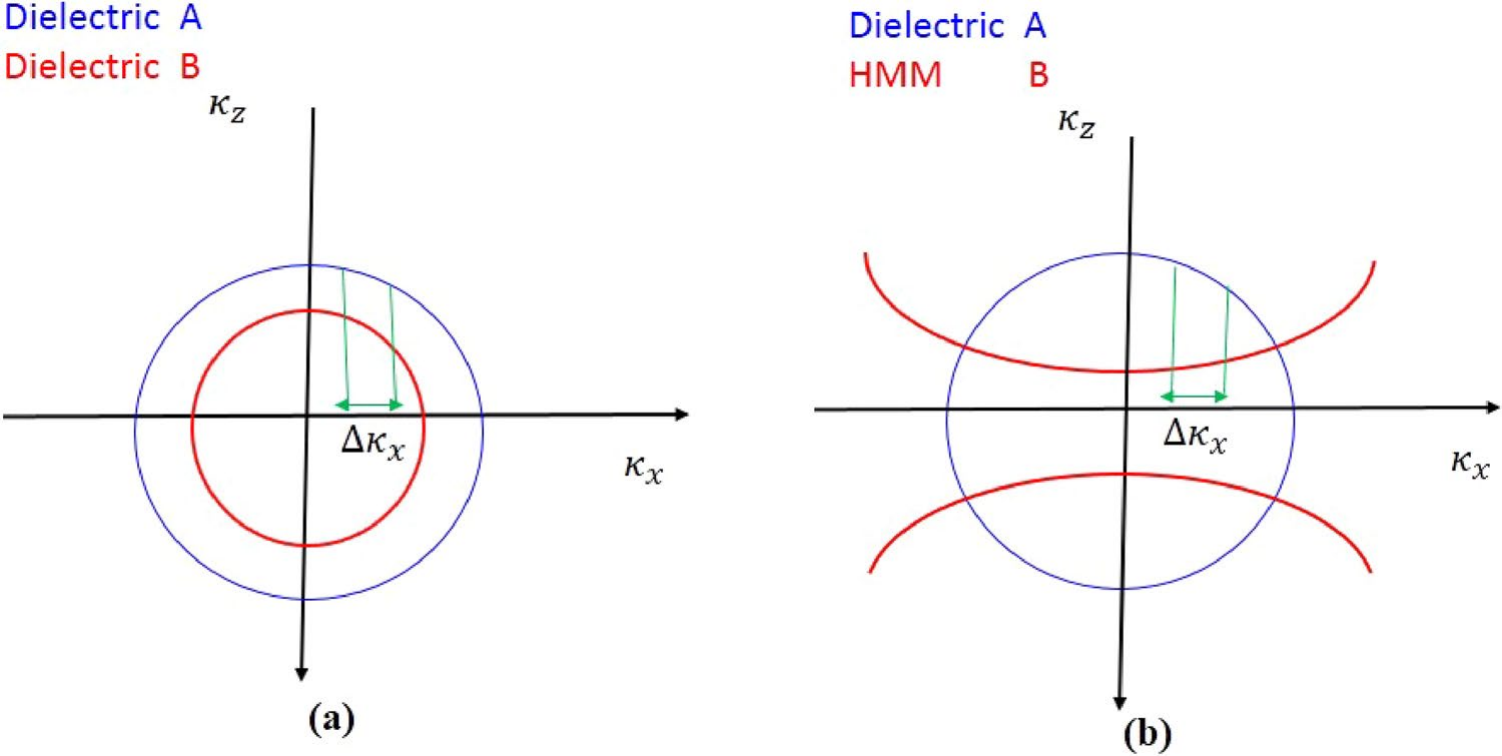
The schematic diagram of the iso-frequency curves of the dielectric and the HMM under TM polarization.

Additionally, the iso-frequency curves of the media in the PC can be used to explain the mechanism. The dielectric layers $A$ and $B$ are specified by their permittivity as $\varepsilon_{A}$ and $\varepsilon_{B}$, respectively. In Fig. 2a, the iso-frequency curves are displayed. The wave vector components $\kappa_{Az}$ and $\kappa_{Bz}$ are simultaneously diminish as the incident angle of the light increases while the horizontal component $\kappa_{x}$ of the incident wave vector is increased. As a result, the obtained PBG of PCs composed of isotropic dielectrics is blueshifted as the incident angle is increased as shown in Fig. 1. Otherwise, as the incident angle for TM waves increases, the PBG becomes narrower. We also take into account a PC with the construction of $\left[ {AB} \right]^{N}$ that contain HMMs where medium A is a typical dielectric material and medium B is a HMM. Figure 2b displays the iso-frequency curves for layers A and B. It was found that as $\kappa_{x}$ is increased, $\kappa_{Bz}$ in layer B is increased while $\kappa_{Az}$ in layer A is decreased. The shift behavior of the band edge in the PC changes as the incident angle increases due to a phase variation compensating effect in the unit cell made up of the HMM and the dielectric layers^36, 37^.

The Bragg scattering approach can account for the PBG in an all-dielectric 1D-PCs blue shift characteristic. It is well known that the wavelength and the incident angle can be used to express the propagating phase of all-dielectric 1D-PC within a unit cell^41^.

1 $${\Phi }\left( {\lambda , \theta } \right) = \kappa_{Az} \left( {\lambda , \theta } \right)d_{A} + \kappa_{Bz} \left( {\lambda , \theta } \right)d_{B}$$

From this equation, $\kappa_{Az}$ and $\kappa_{Bz}$ are standing for the wave vectors (z components) inside the dielectric layers A and B, perpendicular to the interface, respectively. When the Maxwell equations are modified to include the relative permittivity of dielectric layers ($\varepsilon_{A}$ and $\varepsilon_{B}$) under TM polarization^28^, we have the equation of iso-frequency curve (IFC) of dielectric A and B as follows.

2 $$\frac{{\kappa_{x}^{2} }}{{\varepsilon_{A} }} + \frac{{\kappa_{Az}^{2} }}{{\varepsilon_{A} }} = \kappa_{0}^{2} = \left( {\frac{2\pi }{\lambda }} \right)$$

3 $$\frac{{\kappa_{x}^{2} }}{{\varepsilon_{B} }} + \frac{{\kappa_{Bz}^{2} }}{{\varepsilon_{B} }} = \kappa_{0}^{2} = \left( {\frac{2\pi }{\lambda }} \right)$$

where ${\kappa }_{0}$ denotes the wave vector in vacuum and ${\kappa }_{x}$ denotes the wave vector for $x$ component which it is parallel to the interface. The IFCs of dielectric layers A and B are both circular as a result of Eqs. (2) and (3), as schematically depicted in Fig. 2a.

When Eqs. (2) and (3) are then changed to $\kappa_{x} = \kappa_{0} \sin \theta$ we obtain

4 $$\kappa_{Az} = \frac{2\pi }{\lambda }\sqrt {\varepsilon_{A} - \sin^{2} \theta }$$

5 $$\kappa_{Bz} = \frac{2\pi }{\lambda }\sqrt {\varepsilon_{B} - \sin^{2} \theta }$$

The final result can then be obtained by putting Eqs. (4) and (5) into Eq. (1) then, we have the following equation.

6 $${\Phi }\left( {\lambda , \theta } \right) = \frac{2\pi }{\lambda }\left( {d_{A} \sqrt {\varepsilon_{A} - sin^{2} \theta } + d_{B} \sqrt {\varepsilon_{B} - sin^{2} \theta } } \right)$$

Our results from Eq. (6) are $\partial {\Phi }/\partial \lambda < 0$ and $\partial {\Phi }/\partial \theta < 0.$

The lowest-frequency PBG which resulting from Bragg condition can be determined using the Bragg dispersion theory which is given by^42^.

7 $$\Phi \left( {\lambda_{{{\text{Brg}}}} , \theta } \right) = \frac{2\pi }{{\lambda_{{{\text{Brg}}}} }}\left( {d_{A} \sqrt {\varepsilon_{A} - \sin^{2} \theta } + d_{B} \sqrt {\varepsilon_{B} - \sin^{2} \theta } } \right) = \pi$$

where, $\lambda_{Brg}$ is the Bragg wavelength of the lowest-frequency PBG. When $\partial {\Phi }/\partial \lambda < 0$ and $\partial {\Phi }/\partial \theta < 0$, the Bragg wavelength $\lambda_{Brg}$ must decrease as the incident angle increases in order to preserve the Bragg condition (Eq. (7). In all-dielectric 1D-PC, the PBG therefore changes towards shorter wavelengths as the incident angle rises. Likewise, the PBG edges will move toward the shorter wavelength direction.

Therefore, as simply represented by the red solid line in Fig. 2b, the IFC of the HMM is a hyperbola. The $x$ component of the wave vector $\kappa_{x}$ increases as the incident angle increases. As a result, the HMM $\kappa_{Bz}$ wave vector z component increases as well which in turn causing $\partial \kappa_{Bz} /\partial \theta > 0$. In this case, we can get the IFC equation of dielectric layer (A) and HMM layer (B) under TM polarization^28^.

$$\frac{{\kappa_{x}^{2} }}{{\varepsilon_{A} }} + \frac{{\kappa_{Az}^{2} }}{{\varepsilon_{A} }} = \kappa_{0}^{2} = \left( {\frac{2\pi }{\lambda }} \right)$$

8 $$\frac{{\kappa_{x}^{2} }}{{\varepsilon_{Bz} }} + \frac{{\kappa_{Bz}^{2} }}{{\varepsilon_{Bx} }} = \kappa_{0}^{2} = \left( {\frac{2\pi }{\lambda }} \right)$$

Then under TE polarization this equations become as follows.

$$\frac{{\kappa_{x}^{2} }}{{\varepsilon_{A} }} + \frac{{\kappa_{Az}^{2} }}{{\varepsilon_{A} }} = \kappa_{0}^{2} = \left( {\frac{2\pi }{\lambda }} \right)$$

9 $$\kappa_{x}^{2} + \kappa_{Bz}^{2} = \varepsilon_{Bx} \kappa_{0}^{2} = \varepsilon_{Bx} \left( {\frac{2\pi }{\lambda }} \right)$$

The blue solid line in Fig. 2b represents the IFC of the isotropic dielectric graphically where it is a circle as shown in this figure. The $x$ component of the wave vector $\kappa_{x}$ increases as the incident angle rises. As a result, the wave vector z component in the isotropic dielectric $\kappa_{Az}$ drops resulting in $\partial \kappa_{Az} /\partial \theta < 0$. Since $\partial \kappa_{Bz} /\partial \theta > 0$ and $\partial \kappa_{Az} /\partial \theta < 0$, the phase-variation compensation condition must be satisfied for $\frac{{\partial {\Phi }}}{\partial \theta } = 0$ to be realized. By substituting Eqs. (8) and (9) into Eq. (2) we obtain

10 $$\frac{\partial \Phi }{{\partial \theta }} = \frac{{\kappa_{Az} }}{\partial \theta }d_{A} + \frac{{\partial \kappa_{Bz} }}{\partial \theta }d_{B} = 0$$

**Figure 3:**
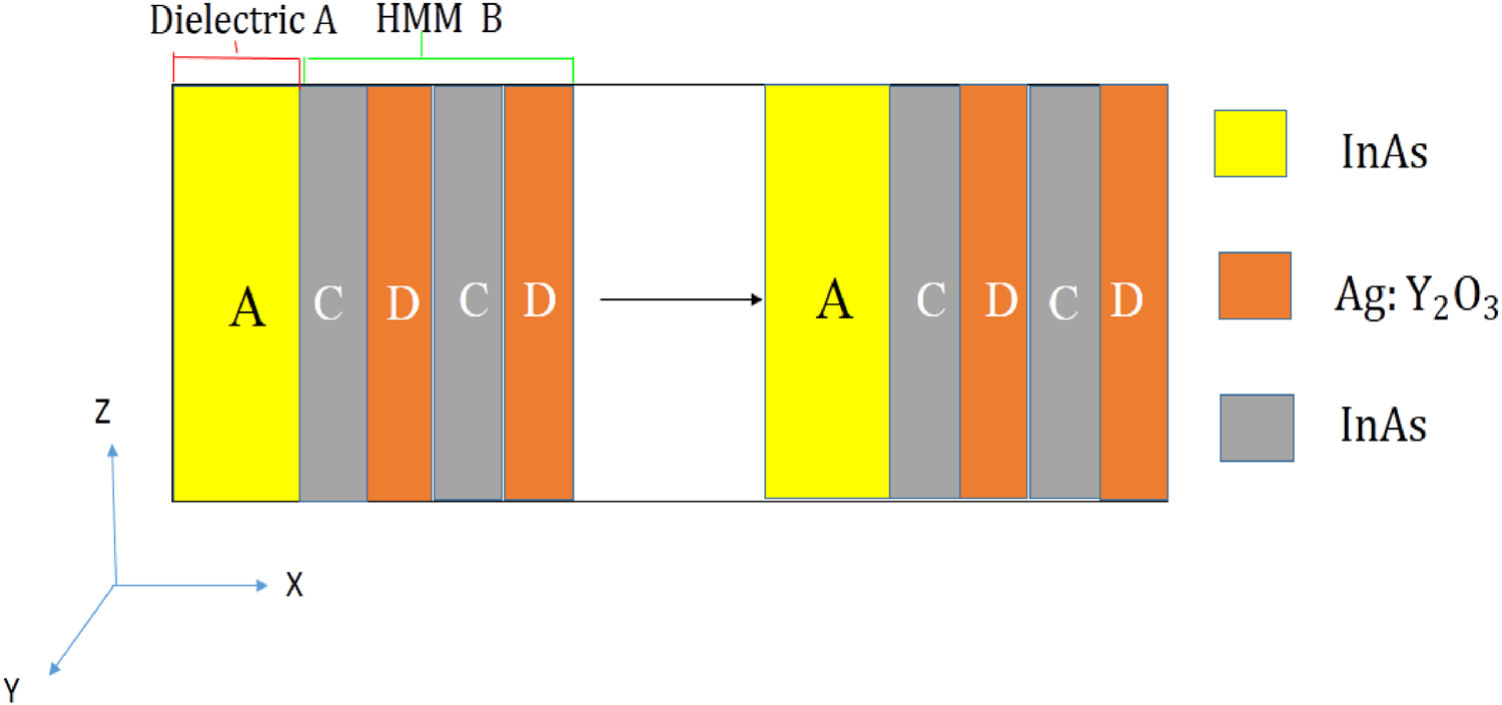
Schematic diagram of PC structure that configured as ${\left[\mathrm{InAs}/{\left(\mathrm{InAs}/\mathrm{ Ag}:{\mathrm{Y}}_{2}{\mathrm{O}}_{3}\right)}^{\mathrm{S}}\right]}^{\mathrm{N}}$ with S = 10, N = 30.

As illustrated in Fig. 3, the HMM layer B in this study is a subwavelength dielectric- nanocomposite multilayer has the configuration of $\left( {CD} \right)^{S}$. In this suggested design, C and D are representing the dielectric material and nanocomposite material, respectively where S is the periodic number. The construction of $\left[ {A\left( {CD} \right)^{S} } \right]^{N}$ is the expression for the structure of the PC $\left( {AB} \right)^{N}$ including HMMs. The dielectric layers A and C containing ${\text{InAs}}$ with the relative permittivity $\varepsilon_{A}$ = $\varepsilon_{c}$ = 3*.*52^43^ and layer D is the nanocomposite layer which consisted of ${\text{Ag}}$ nanoparticles emerged into host material ${\text{ Y}}_{{2}} {\text{O}}_{{3}}$. The Maxwell–Garnett model is used to determine the effective permittivity ($\varepsilon_{D}$) of silver nanoparticles implanted in ${\text{Y}}_{2} {\text{O}}_{3}$ where it is given by the following equation:

11 $$\varepsilon_{D} = \frac{{2\varepsilon_{b} f\left( {\varepsilon_{m} - \varepsilon_{b} } \right) + \varepsilon_{b} \left( {\varepsilon_{m} + 2\varepsilon_{b} } \right)}}{{2\varepsilon_{b} + \varepsilon_{m} + f\left( {\varepsilon_{b} - \varepsilon_{m} } \right)}}$$

where $\varepsilon_{b}$, $\varepsilon_{m}$ and $f$ are representing, Ag–NPs permittivity, host material permittivity, and the filling fraction of NPs, respectively. The Drude model is then used to compute the permittivity of Ag-NPs^44^ as follows.

12 $$\varepsilon_{m} = \varepsilon_{0} - \frac{{\omega_{p}^{2} }}{{\omega^{2} + i\omega \delta }}$$

where $\varepsilon_{0}$, $\delta$ and $\omega_{p}$ refers to the high frequency limit of permittivity, damping frequency, and Plasmon frequency, respectively. Then, the damping frequency is decreased if the radius of NPs is increased according to the following equation:-

13 $$\delta \left( r \right) = \delta_{0} + \frac{{{\text{V}}_{f} }}{r}$$

where, ${\text{V}}_{f}$ is the electron velocity at Fermi energy and $\delta_{0}$ denotes the decay constant.

In our calculations, the values of the parameters are chosen to be $\omega_{p} = 2\pi \times 2.17 \times 10^{15} {\text{Hz}}$ and $\delta_{0} = 2\pi \times 4.8 \times 10^{12} {\text{Hz}}$ and $\varepsilon_{0} = 5$ respectively^45–47^.

The effective relative permittivity tensor of the subwavelength ${\text{InAs}}$/ ${\text{Ag}}:{\text{Y}}_{2} {\text{O}}_{3}$ multilayers $\left( {CD} \right)^{10}$ can be written using the effective medium theory^28^ as follows.

14 $$\varepsilon_{B} = \left[ {\begin{array}{*{20}c} {\varepsilon_{Bx} } & {\quad 0} & {\quad 0} \\ 0 & {\quad \varepsilon_{Bx} } & {\quad 0} \\ 0 & {\quad 0} & {\quad \varepsilon_{Bz} } \\ \end{array} } \right]$$

where,

15 $$\varepsilon_{Bx} = p\varepsilon_{C} + \left( {1 - p} \right)\varepsilon_{D}$$

16 $$\frac{1}{{\varepsilon_{Bz} }} = \frac{p}{{\varepsilon_{C} }} + \frac{{\left( {1 - p} \right)}}{{\varepsilon_{D} }}$$

**Figure 4:**
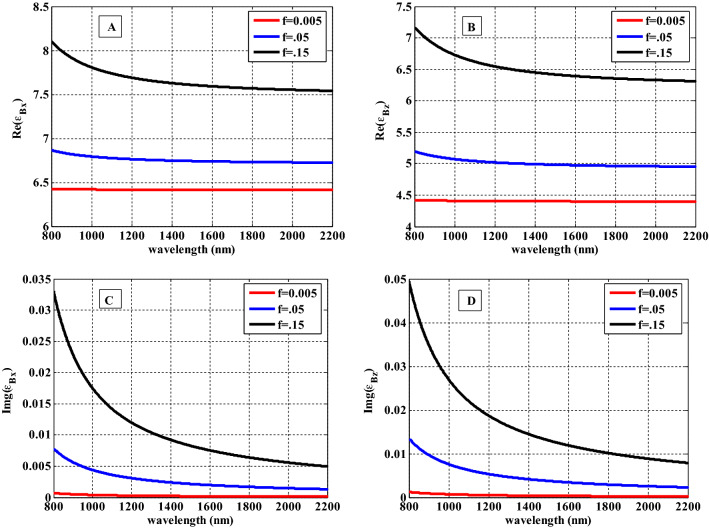
Effective permittivity of HMM (Real and imaginary parts) in the x and z components as a function of wavelength.

The filling ratio of the subwavelength ${\text{InAs}}$ layer within the HMM is shown here as $p = \frac{{d_{C} }}{{d_{C} + d_{D} }}$. In the structure, $p = 0.35$ is chosen. We determine the $x$ and $z$ components of the subwavelength multilayer ${\text{InAs}}$/ ${\text{Ag}}:{\text{Y}}_{2} {\text{O}}_{3}$ $\left( {CD} \right)^{10}$ due to effective relative permittivity tensor as a function of wavelength using Eqs. (15) and (16) as shown in Fig. 4. Figure 4 indicates the response of the effective relative permittivity of HMM $\left( {CD} \right)^{10}$ through the wavelength range from 800 to 2200 nm. From this figure, we observed that the real part of ${\text{Re}} (\varepsilon_{Bx} )$ is greater than that of ${\text{Re}} (\varepsilon_{Bz} )$ where both of them are increasing with changing the filling fractions of ${\text{Ag}}:{\text{Y}}_{2} {\text{O}}_{3}$ to values of $f = 0.005$, $f = 0.05,$ and $f = 0.15$. Both the wavelength of incident radiation and the filling fraction of the nanoparticles have a significant impact on both the real parts [${\text{Re}} \left( {\varepsilon_{Bx} } \right),{\text{Re}} \left( {\varepsilon_{Bz} } \right)$] and the imaginary parts [${\text{Im}} g(\varepsilon_{Bx} ), \;{\text{Im}} g(\varepsilon_{Bz} )$] of permittivity components. The ${\text{Re}} \left( {\varepsilon_{Bx} } \right)$ and ${\text{Re}} \left( {\varepsilon_{Bz} } \right)$ are significantly affected by increasing the filling fraction as shown in Fig. 4A and B. Based on Eq. (11), such effect might be simply clarified. This occurs as a result of an increase in Ag-NPs concentration in the host dielectric material. As a result, the dispersion properties of the permittivity of HMM material become prominent. The interaction of the incident electromagnetic waves with the surface plasmon of Ag-NPs leads to the formation of an asymmetrical mode for both real and imaginary portions that is concentrated at a specific wavelength region^48^. Figure 4C and D show that the imaginary parts ${\text{Im}} g(\varepsilon_{Bx} )$ and ${\text{Im}} g(\varepsilon_{Bz} )$ are close to zero and independent of the wavelength ranges of incident radiation.

The EMWs response through PCs was simply explored using the transfer matrix method^49, 50^. The following matrix equation can be used to specify our design,

17 $$M_{{{\text{structure}}}} = \left( {\begin{array}{*{20}c} {M_{11} M_{12} } \\ {M_{21} M_{22} } \\ \end{array} } \right) = \left( {M_{A} M_{B} } \right)^{N}$$

The characteristic matrices for the dielectric layer (layer A) and the HMM layer (layer B) are $M_{A}$ and $M_{B}$, respectively where:

18 $$M_{j} = \left( {\begin{array}{*{20}c} {\cos \gamma_{j} - \left( {i/q_{j} } \right)\sin \gamma_{j} } \\ { - iq_{j} \sin \gamma_{j} \cos \gamma_{j} } \\ \end{array} } \right) = \left( {M_{A} M_{B} } \right)^{N}$$

With, $\left( {\gamma_{j} = \frac{{2\pi d_{j} }}{\lambda }n_{j} \cos {\uptheta }_{j} ,j = 1,2} \right),\left( {q_{j} = \sqrt {\frac{{\varepsilon_{0} }}{{\mu_{0} }}} n_{j} /\cos \theta_{j} ,j = 1,2} \right)$ and $\theta_{j}$ is the incident angle within layer $j$. The transmission coefficient can be determined using components of the matrix in Eq. (17) as follows.

19 $$t = \frac{{2p_{0} }}{{\left( {M_{11} + M_{12} p_{s} } \right)p_{0} + \left( {M_{21} + M_{22} p_{s} } \right)}}$$

20 $$p_{0,s} = \sqrt {\frac{{\varepsilon_{0} }}{{\mu_{0} }}} n_{0,s} \cos n_{0,s}$$

Then, the transmittance can be expressed as follows.

21 $$T = \frac{{p_{s} }}{{p_{0} }}\left| {t^{2} } \right|$$

## The optical properties of the proposed PC design containing HMM

The suggested structure is consisting of alternating dielectric material and HMM layers where the first layer is ${\text{InAs}}$ (as a layer A) with $d_{1} = 50 {\text{nm}}$ and the second layer is the HMM (dielectric/ NC) ${\text{InAs}}$/ ${\text{Ag}}:{\text{Y}}_{2} {\text{O}}_{3}$ (as a layer B) with $d_{2} = 1500 {\text{nm}}$. The values of the Ag–NPs radius and the filling fraction of the composite are $r = 30 {\text{nm}}$ and $f = 0.005$, respectively. In this case, we consider the periodicity number of our suggested structure to be (N) = 30.

**Figure 5:**
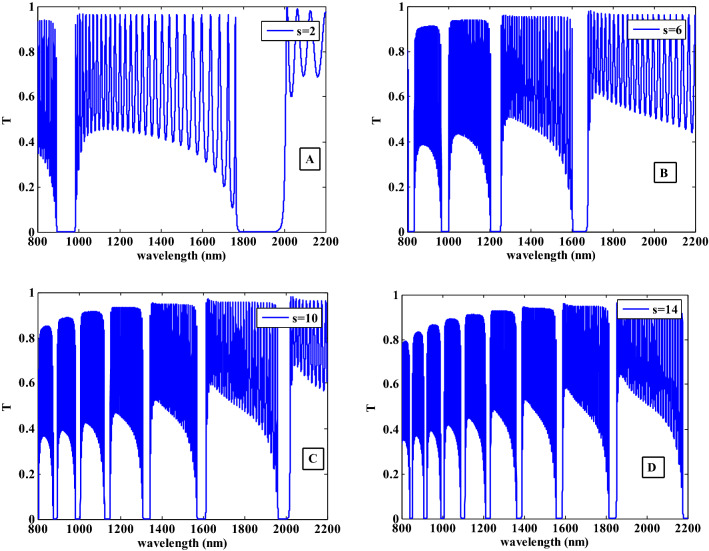
The transmittance characteristics of the proposed one-dimensional photonic crystal design containing HMM at different S (periodicity of HMM).

By changing the period of HMM layer inside the proposed structure, we observed that the number of resulted PBGs will change as shown in Fig. 5. In Fig. 5A, when the periodic number of HMM has the value of S = 2, we obtained two PBGs. Then, with increasing S = 6, 10 and 14 as seen in Fig. 5B–D, the number of PBGs are increased. From Fig. 5, we observed that the width of the PBGs are decreased and it became sharper. The increase in the periodicity of HMM meaning that an increase in the thickness of this layer. As a result, with increasing the HMM layer thickness, new gaps begin to form at lower wavelengths and the number of them will increase that can be useful in some applications like the optical channel filter. In the following studies, we fixed the value of periodicity of HMM at S = 10.

As shown in Fig. 3, the proposed design is sandwiched between a substrate and air as a beginning medium that configured as $\left[ {{\text{InAs}}/\left( {{\text{InAs}}/{\text{Ag}}:{\text{Y}}_{2} {\text{O}}_{3} } \right)^{10} } \right]^{30}$. The single material HMM is mimicked by a subwavelength of dielectric layer and nanocomposite ${\text{InAs}}$/ ${\text{Ag}}:{\text{Y}}_{2} {\text{O}}_{3}$ multilayer $\left( {CD} \right)^{10}$. In our calculations, the filling ratio of ${\text{ InAs}}$ layer is *p* = 0*.3*5. As a result, we can obtain $d_{C} = 52.5 {\text{nm}}$, $d_{D} = 97.5 {\text{nm}}$. The thickness of the unit cell of the HMM layer is $d = 150 {\text{nm}}$ (about 0.031 times of the designed Bragg wavelength) which ensures the accuracy of the effective medium theory^51^.

Based on the prior analysis, the optical features of the proposed design have been introduced in the following sections. Meanwhile, we studied the transmittance properties of binary 1D-PCs containing HMM. We showed how the host material permittivity and the incidence angle of EMW for TM and TE polarization can affect the suggested PCs transmittance characteristics. In addition, the effect of the thickness of the HMM layer $d_{2}$ and the filling fraction of NC material inside HMM have been demonstrated on the transmittance characteristics of the proposed PC design.

**Figure 6:**
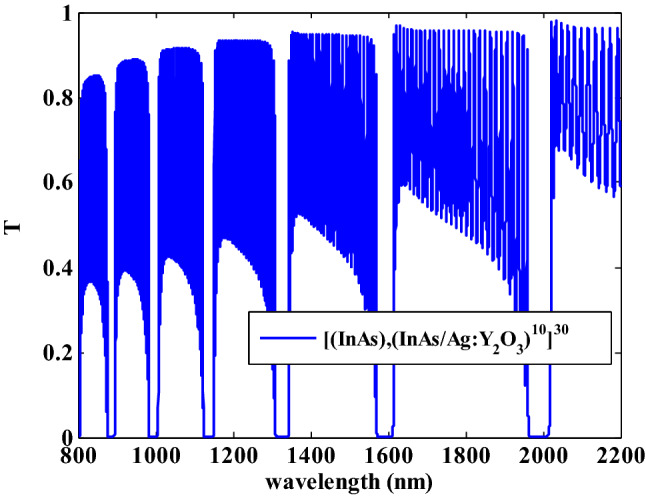
The transmittance spectra of the proposed one-dimensional photonic crystal containing HMM.

Figure 6 demonstrates the transmittance spectra of the suggested PC design containing the dielectric and HMM materials NIR region. From this figure, we noticed that there are six Bragg PBGs with different widths in the considered wavelength spectrum. It is worth mentioning that the Bragg PBGs occurred as a result of the Bragg condition and the interaction of the electromagnetic waves in the interfaces between the constituents layers^52^. Also, the PBGs is the result of a significant difference in refractive indices between the two constituent’s layers of the proposed design. In addition to that, this PBG appearance can be ascribed to the constructive interference of the reflected waves at the interface between various layers^53^. Compared to the outcomes displayed in Fig. 4, the real effective permittivity components ${\text{Re}} (\varepsilon_{Bx} ) \;{\text{and}} \;{\text{Re}} (\varepsilon_{Bz} )$ are changed to 6.245 and 4.452 at filling fraction $f = 0.005$ NIR range. Furthermore, the imaginary effective permittivity components for the same wavelengths are close to zero. The response of the effective permittivity of the HMM layer at these wavelengths NIR range is causing PBGs to occur in this region.

**Figure 7:**
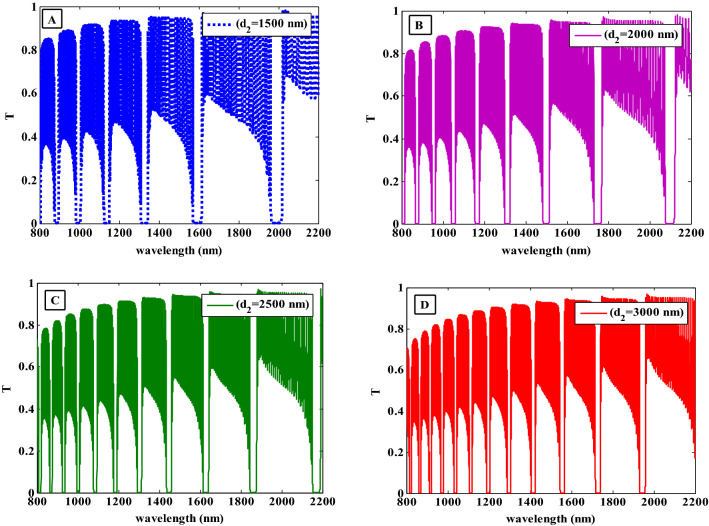
The transmittance spectra of the suggested one-dimensional photonic crystal design at different thickness of HMM layer.

Figure 7 displays the transmittance characteristics of the proposed PC at various HMM layer thicknesses such as 1500 nm, 2000 nm, 2500 nm, and 3000 nm with fixing the values of the radius and filling fraction of the Ag-NPs and the dielectric layer thickness at r = 30 nm, $f = 0.005$ and $d_{1} = 50 {\text{nm}}$, respectively. Figure 7 illustrates that the PBGs characteristics are significantly influenced by the HMM layer thickness. As the HMM layer thickness is increased to the values of 1500, 2000, and 2500 nm, the number of PBGs are increased to six, eight, and ten as shown in Fig. 7A–C, respectively. Also, there is a shift towards the higher wavelengths and the bandwidth is gradually dropped. The position of the PBGs is pushed towards the higher wavelengths areas with decreasing their width. In addition, the number of PBGs are increased to twelve as the thickness of the HMM layer is increased to 3000 nm as seen in Fig. 7D. As a result, with increasing the HMM layer thickness, new gaps begin to form at lower wavelengths. Additionally, the PBGs are shifted to higher wavelengths as the thickness is increased according to the following equation^54^:

22 $$\varphi_{i} = 2\pi n_{i} d_{i} \cos \left( {\theta_{i} } \right)/\lambda$$

where $\varphi$ stands for the phase condition, $n$ refers to the refractive index of a layer, $d$ indicates to the layer thickness, $\theta$ is the incident angle and $\lambda$ is the wavelength. Equation (22) states that this shift in PBGs occurs when the thickness of the HMM layer is increased because with fixed refractive index $n$ and $\cos \left( \theta \right)$ values, the wavelength $\lambda$ is increased.

Additionally, even if the value of the filling fraction is constant, the width of the PBGs is gradually decreased and the width of the new PBGs is gradually increased due to an increase in the interaction of the EMW with the HMM layer as more Ag-NPs are put into the NC material^55^. As a result, the thicknesses of the PCs constituent materials can be used to change the widths, positions, and quantity of PBGs. The obtained PBGs in our proposed structure could be used in beneficial applications like a multichannel filter.

**Figure 8:**
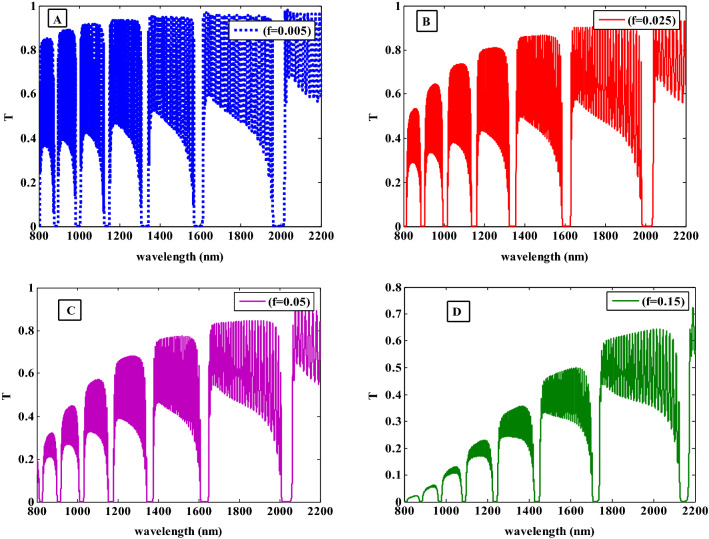
The transmittance spectra of the one-dimensional photonic crystal containing HMM at different filling fractions of $\mathrm{Ag}:{\mathrm{Y}}_{2}{\mathrm{O}}_{3}$ .

In the following study, we will investigate the filling fraction effects on the resulted optical transmittance of the proposed structure. The relationship between the PBGs width, positions, and filling fraction of Ag-NPs is presented in Fig. 8. The effective permittivity of the HMM layer is significantly affected by modifying the filling fraction, especially in the real part as illustrated in Fig. 4. This might be caused by Ag-NPs dispersion characteristics which start to become prominent at high filling fractions. With increasing the filling fraction from $0.005$ (see Fig. 8A) to $0.025$ and $0.05$ (see Fig. 8B and C), the PBGs is somewhat moved to higher wavelengths in comparison to the findings of the study in Fig. 6. Also, the transmittance oscillations are decreased due to increasing the Ag-NPs with increasing the filing fraction. Additionally, a new PBG started to appear at lower wavelengths which is the reason for the width of PBGs increasing as seen in Fig. 8B and then decreases as shown in Fig. 8C. This response is directly connected to the change in the number of Ag-NPs as the filling fraction changes, i.e., the width of PBG increases as a result of an increase in the interaction between the EMW and structure caused by an increase in Ag-NPs as the filling fraction increases. Moreover, in Fig. 8D, the width of PBGs and the transmittance are more decreased with increasing filling fraction to $f = 0.15$. The HMM effective permittivity is changed to greater values as seen in Fig. 4 which may be the cause of this response to the position and width of the PBGs.

**Figure 9:**
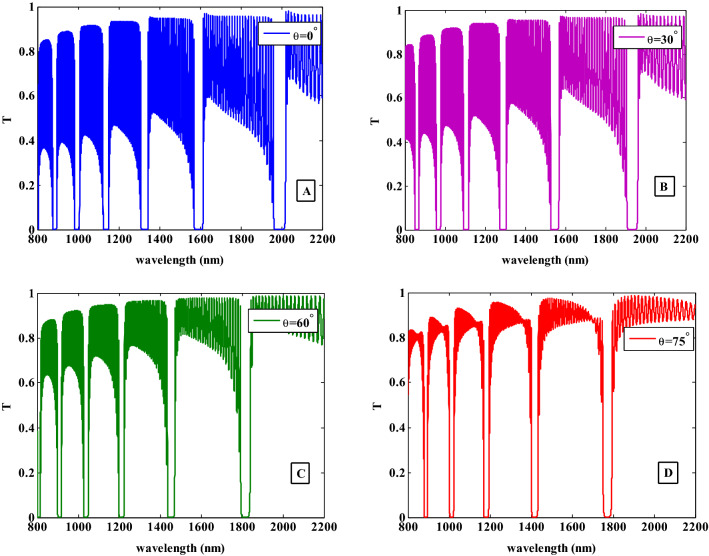
The transmittance spectra of the one-dimensional photonic crystal containing HMM at different incident angles for TM polarization.

Here, we demonstrate the effects of the angle of incidence on the transmittance spectra of the suggested PC design for TM polarization as indicated in Fig. 9. In our calculations, the incident angle changes from $\theta = 0^{^\circ }$ to $\theta = 30^{^\circ }$ , $\theta = 60^{^\circ }$ and $\theta = 75^{^\circ }$ at $r = 30\;{\text{nm}}$ and $f = 0.005$. As seen in Fig. 9, the increase in the angle of incidence up to 75° has a significant impact on the transmittance values. This indicates that the oscillation of the transmittance outside the PBGs is decreased with increasing the incident angle. Also, the number of PBGs in the transmittance spectra has been decreased where it shifted downwards the lower wavelengths and its width has been decreased with increasing the incident angle. Figure 9C and D present the transmittance levels for this wavelength range have significantly decreased. This result is given by Eqs. (19) and (20) which show the connection between transmission and incident angle. We found that the transmission is reduced as the angle of incidence is increased.

Additionally, the shift of the PBGs can be explained by the Bragg-Snell law which is given by the subsequent equation,

23 $$m\lambda = 2D\sqrt {n_{eff}^{2} - \sin^{2} (\theta )}$$

It can be utilized to describe how the PBGs behaviour changes when the incident angle rises^56–59^. In this equation, *n*_*eff*_ is the effective refractive index, *m* is the diffracted order, D is the interplanar spacing, $\theta$ is the incident angle, and $\lambda$ is the wavelength of light. This equation demonstrates that the incident angle and the incident wavelength are inversely related, and the increase in the incident angle causes the PBG to move to lower wavelengths.

**Figure 10:**
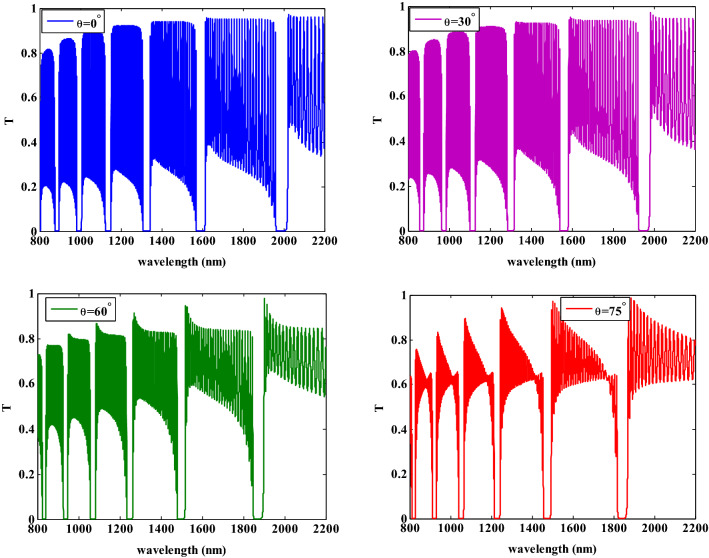
The transmittance spectra of the one-dimensional photonic crystal containing HMM at different incident angles for TE polarization.

Similar to the study in Fig. 9, we have investigated the transmittance characteristics for the TE mode at the same incident angles, radius, filling fraction, and thicknesses of layers. As the incident angle increases, the PBGs are shifted to the lower wavelengths and its width is decreased. For a comparison between TE and TM modes in Figs. 9 and 10, we observed that the PBGs are more sensitive for TM mode than TE mode i.e. TE mode shows a higher reflection than TM mode at higher incident angle values.

**Figure 11:**
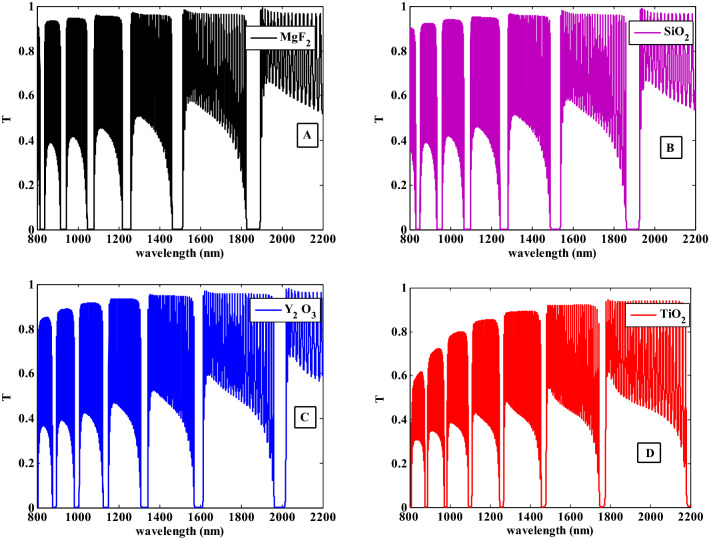
The transmittance spectra of the one-dimensional photonic crystal having HMM at different host materials.

Then, we examined the effects of the permittivity of the host dielectric material on the PBGs characteristics where the transmittance spectra are shown in Fig. 11. Here, we used different dielectric materials such as ${\text{MgF}}_{2}$, SiO2, ${\text{Y}}_{2} {\text{O}}_{3}$, and ${\text{TiO}}_{2}$ with permittivities of $1.92$, $2.25$, $3.22$, and $5.52$, respectively, at filling fraction $f = 0.005$. Furthermore, the filling ratio of supwavelengths layer and the spherical radius of Ag–NPs have been fixed to $p = 0.35$ and $r = 30\; {\text{nm}}$. As illustrated in Fig. 11, the position of the PBGs is shifted towards the higher wavelengths with increasing the permittivities of the host materials. In addition to that, there are new PBGs are beginning to appear as shown in Fig. 11D. By using different host dielectric materials, the effective permittivity of the NC material and HMM layer will change according to the Eqs. (11) and (14). As a result, the host material permittivity could be used to control the obtained PBGs.

**Figure 12:**
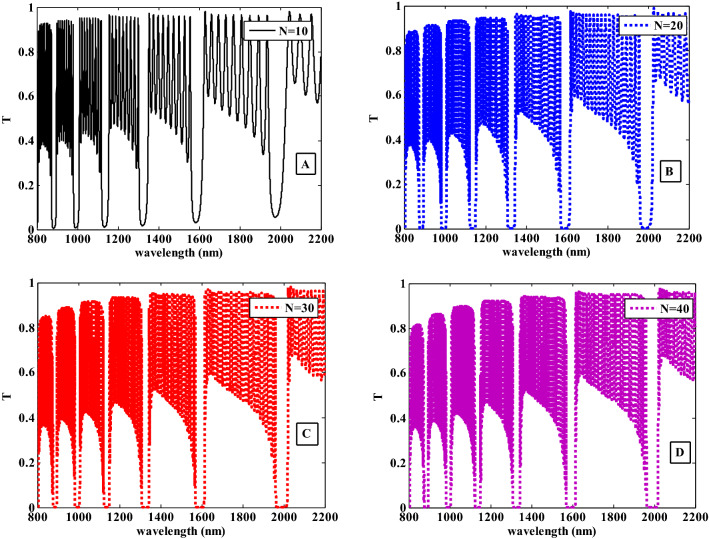
The transmittance spectra of the one-dimensional photonic crystal containing HMM at different values of the number of periods of the structure (N).

Figure 12A–D illustrate the resulted transmittance spectra at different values for the structure number of periods. From these figures, we observed that, as the number of period increases, the PBG edges become more sharp and steeper. This behavior can be explained and described by the following equation.

24 $$L = N\Lambda$$

In this equation, $L$ is the overall length of the structure, $N$ is the number of periods, and $\Lambda$ is the structure's lattice constant where $\Lambda ={d}_{1}+{d}_{2}$. From Eq. (24), as the number of period increases, the structure's overall length will increase as well. Also, this will increase the number of interfaces and the possibility that the electromagnetic waves inside the structure will be absorbed and reflected^60, 61^. As a result, the PBG edges become more sharp and steeper as shown in Fig. 12A–D.

## Conclusion

To sum up, we have demonstrated the optical properties of a 1D-PC structure containing hyperbolic metamaterials (HMMs) for optical filtering purposes. The Maxwell–Garnett model and the transfer matrix method are used for the theoretical verifications. The management of the various PBG parameters can be controlled from the numerical investigations of the dispersion properties of the HMM layer. The angle-dependent omnidirectional PBG is demonstrated in this study in a PC structure made of dielectric materials and HMMs created by subwavelength dielectric-NC multilayers. Variation of the design factors such as HMM layer thickness, background material permittivity, filling fraction and the incident angle for TM and TE polarization can also regulate the position and the width of the obtained PBG. The proposed structure has the potential to significantly improve the quality and the efficiency of the aforementioned optical and photonic fields as it can be used in enhancing the properties of optical multi-channel filters.

## Data Availability

The datasets used and/or analyzed during the current study are available from the corresponding author on reasonable request.
